# Too much of a good thing? The moderating role of children’s perceived social support in drawing activities

**DOI:** 10.1371/journal.pone.0330470

**Published:** 2026-02-17

**Authors:** Taiyu Nie, Kuan-Chun Tsai, Jun Wu

**Affiliations:** 1 Faculty of Education, Ningxia Normal University, Guyuan, China; 2 Chinese International College, Dhurakij Pundit University, Bangkok, Thailand; 3 School of Art and Design, Division of Arts, Shenzhen University, Shenzhen, China; PLOS: Public Library of Science, UNITED KINGDOM OF GREAT BRITAIN AND NORTHERN IRELAND

## Abstract

Art engagement plays a crucial role in children’s flourishing, yet there is limited understanding of how individual traits and environmental factors shape children’s art engagement. Drawing on Cognitive Evaluation Theory (CET), this study examines the relationship between children’s drawing self-efficacy and art engagement, as well as the moderating role of perceived social support. A cross-sectional survey was conducted with 592 children aged 10–12 in Henan Province, China, using paper-based questionnaires. The results indicated that both drawing self-efficacy and perceived social support positively predicted art engagement; however, high levels of perceived social support weakened the positive relationship between drawing self-efficacy and art engagement. These findings challenge the common assumption that social support is uniformly beneficial and highlight the importance of autonomy-supportive practices. The key challenge lies in providing support that strengthens children’s autonomy in drawing activities. Parents and art educators should therefore adopt autonomy-supportive approaches to help children remain actively engaged in drawing activities and achieve long-term developmental benefits.

## 1. Introduction

In the compulsory education stage, visual arts education plays an irreplaceable role in children’s comprehensive development [1–3]. As a core component of visual arts education, drawing is not only an important medium through which children express themselves and explore the world, but also a foundational and representative visual art activity in primary education, fostering creativity, aesthetic ability, and problem-solving skills [4, 5]. Previous research has shown that drawing activities help children master the use of artistic tools while also promoting emotional expression, life reflection, and the development of unique artistic language [6]. Moreover, engagement in drawing as a hands-on activity has a significant impact on children’s cognitive development, emotional growth, and socialization processes [7–9].

These benefits collectively constitute the core dimensions of art engagement, including immersion, self-expression, reflection, acquisition, and socialization [10]. Specifically, immersion refers to a state of deep concentration and effortless involvement in the drawing process. Self-expression denotes the use of drawing to convey emotions, articulate thoughts, and communicate perspectives. Reflection represents a conscious process with both cognitive and affective components, through which children review, adjust, or reinforce their habits, values, or worldviews. Acquisition captures the ongoing process of applying, developing, and refining drawing skills. Finally, socialization highlights the interpersonal aspects of drawing, such as interaction with peers, understanding others, and enhancing self-awareness [11]. Through art engagement, parents and art educators can gain deeper insights into children’s subjective experiences and psychological involvement in artistic activities [12, 13].

However, children’s engagement in artistic activities is uneven and influenced by both individual characteristics and environmental support [14, 15]. Within the context of visual arts activities, drawing self-efficacy represents a key individual psychological factor [16–20], while perceived social support functions as an external factor that shapes children’s artistic experiences and promotes enjoyment and persistence [21–25]. Importantly, social support is not always beneficial. When perceived as controlling, such as through excessive rewards or forced participation, it may undermine children’s intrinsic motivation and, in turn, reduce their art engagement [26].

Although existing studies have emphasized the roles of individual traits and environmental support in shaping art engagement, systematic empirical research examining the relationship between drawing self-efficacy and art engagement remains limited [19]. Moreover, support mechanisms for children’s art engagement within family, school, and broader social contexts have not been fully explored [8,27]. Prior research has often focused on broad indicators, such as participation frequency and activity type, while paying relatively less attention to children’s subjective experiences and psychological involvement in art engagement [28, 29]. Such macro-level measurement approaches may obscure the depth and quality of children’s engagement in drawing activities [11,30]. Consequently, current evidence provides an incomplete understanding of how drawing self-efficacy and perceived social support jointly influence children’s art engagement. In particular, few studies have examined perceived social support as a moderating factor in this process.

Therefore, this study aims to examine the relationships among drawing self-efficacy, perceived social support, and art engagement, with a specific focus on the moderating role of perceived social support. This study aims to provide theoretical support for children’s art education and offer practical guidance for families and schools in engaging children in drawing activities.

## 2. Literature review

### 2.1. Drawing self-efficacy and art engagement

Self-efficacy refers to an individual’s belief in their ability to perform specific tasks, which plays a crucial role in motivation, emotional regulation and behavioral decision-making [31]. In the field of drawing, drawing self-efficacy is defined as an individual’s self-evaluation of their own drawing ability, which specifically includes three dimensions: (i) Drawing skills – the ability to draw specific things; (ii) Conveying and communicating – expressing and exchanging ideas to others through drawing; (iii) Creative expression – showcasing imagination and creativity in the process of drawing [32].

Previous studies have shown that self-efficacy significantly affects an individual’s engagement in behavior, emotion, cognition and autonomy [33–35]. Individuals with high self-efficacy are more inclined to actively participate in tasks and demonstrate stronger persistence when faced with challenges [19,20,31]. In visual arts activities, students with high self-efficacy are more confident in their artistic and drawing abilities, thereby enhancing their motivation to participate in artistic activities [8,18,36]. On the contrary, children with low self-efficacy often lack confidence and may avoid or passively participate in drawing activities [17], which may limit the positive role of drawing in cognitive, emotional and social development [37, 38].

Most existing studies have focused on general self-efficacy [8,19,38,39], with limited use of tools to assess children’s drawing self-efficacy. This gap risks overlooking the domain-specific features of drawing in explaining art engagement. Since context-specific self-efficacy is more predictive of actual participation than general beliefs [40], it is necessary to clarify the link between drawing self-efficacy and art engagement in visual art activities [20,39].

### 2.2. Perceived social support and art engagement

Family and school are indispensable environments for children’s growth, and they have a profound impact on their artistic experience and drawing behavior 26. Social support not only helps individuals cope with challenges, but also provides important resources for achieving their goals [41, 42]. Social support from teachers, parents and peers is considered an important factor affecting children’s drawing behavior and art engagement [8,38].

Existing research indicates that the importance, expectations, and attitudes of parents and teachers toward drawing can significantly influence children’s artistic development and modes of self-expression [43]. Individuals who perceive higher levels of social support also tend to engage more actively in a variety of artistic activities [24, 25]. In particular, within a group art creation environment, social support fosters the development of social connections and a sense of belonging [2], and these positive effects are closely related to art engagement. Specifically, teachers’ emotional and ability support significantly enhances students’ sense of belonging and competence [44]. Primary school students who perceive more support from teachers and peers tend to be more actively engaged in emotional, cognitive and behavioral participation in visual arts extracurricular activities [45]. The support of parents is equally crucial. When parents can understand their children’s interests and actively participate in their artistic activities, children can gain more emotional and cognitive benefits in artistic creation [13,46].

However, most studies tend to examine the roles of teachers, peers, and parents separately, with limited attention to social support as an integrated environmental system [11]. This fragmented perspective overlooks potential interactions among different sources of support and constrains understanding of the overall effects of social support. Therefore, revealing the systemic role of social support is a key step toward deepening the understanding of children’s art engagement.

### 2.3. The moderating effect of perceived social support

Social support, especially from teachers, parents and peers, is an important factor affecting children’s engagement in the arts [43,47]. Positive feedback from teachers and encouragement and participation from parents significantly enhance children’s artistic self-efficacy and sense of achievement [8,13,48]. When children have a clear interest in specific activities and receive genuine support, they typically exhibit higher levels of art engagement because this support enhances their intrinsic motivation and confidence [49,50]. However, when social support does not align with children’s mastery goal orientation or artistic interests [51–53], it may be perceived as a form of control. Such controlling support can weaken children’s intrinsic motivation and, in turn, reduce their art engagement [54]. These findings indicate that social support has both facilitative and constraining effects, highlighting the need for a more nuanced analysis of its mechanisms.

Based on the Cognitive Evaluation Theory, social support enhances an individual’s intrinsic motivation by meeting their basic psychological needs, particularly their sense of competence and autonomy [55]. When social support is perceived by individuals as affirmation of their sense of competence and autonomy, it enhances their intrinsic motivation, further strengthening the positive impact of drawing self-efficacy on art engagement [11,36,48]. However, when social support is perceived as external control, such as excessive rewards or forced participation, it may lead to external causal attribution, thereby weakening an individual’s intrinsic motivation [54,56]. In addition, the impact of art education experience on children’s social and emotional development may also be moderated by individual cognitive differences [7]. Nevertheless, existing research on children’s visual art practice has largely focused on the direct effects of different sources of social support, with limited attention to its moderating role in the relationship between self-efficacy and art engagement. In addition, most of these studies are based on Western cultural contexts, leaving the unique variations arising from individual differences and Chinese cultural settings insufficiently explored [27,57,58].

Therefore, the present study positions perceived social support as a moderator of the relationship between drawing self-efficacy and art engagement. This perspective allows for a deeper examination of the dual mechanisms of social support and addresses gaps in both the mechanistic understanding and methodological integration of prior research. Ultimately, these insights can inform the development of a visual arts education support framework that accommodates cultural diversity and individual differences.

### 2.4. Cognitive evaluation theory

Cognitive Evaluation Theory (CET) was proposed by Deci and Ryan and is a core component of Self-Determination Theory (SDT) [55]. It mainly explores the role of social environmental factors in promoting or weakening an individual’s intrinsic motivation. CET emphasizes that satisfying the basic psychological needs of competence, autonomy, and relatedness is essential for fostering intrinsic motivation and enhancing engagement [55]. Competence needs refer to the sense of efficacy that individuals feel during the process of completing tasks, such as when children perceive that they can effectively use drawing techniques to express their ideas; Autonomy needs refer to individuals’ perception of their choices and control over behavior, which in drawing activities is reflected in children’s freedom to select themes, materials, or styles of their artwork; Relatedness needs refer to an individual’s sense of attachment to others, which in drawing can be observed when children share their artwork, receive encouragement, or collaborate on creative tasks. When these needs are met, individuals are more inclined to participate in activities out of intrinsic interest rather than external rewards [55]. However, external rewards weaken intrinsic motivation, especially when rewards are seen as a means of control [54].

Under the CET framework, drawing self-efficacy reflects an individual’s evaluation of their own drawing ability as a manifestation of their competence and autonomy needs [31]. When individuals believe they have the ability to draw, they are more inclined to act independently and experience inner interest and enjoyment in the creative process, which helps to enhance art engagement [34, 35]. In addition, social support plays an important role as an external environmental factor in the CET framework. When social support is perceived by individuals as recognition of their abilities and autonomy, it can enhance their intrinsic motivation and further promote art engagement [36]. However, when social support is seen as a means of control, it may weaken intrinsic motivation [54].

### 2.5. The present study

This study adopts Cognitive Evaluation Theory (CET) as the theoretical framework to examine how children’s drawing self-efficacy influences their art engagement, and to test whether perceived social support moderates this relationship. Based on the literature review, the current study proposes the following hypotheses:

H1: Children’s Drawing self-efficacy is positively associated with art engagement.

H2: Children’s Perceived social support is positively associated with art engagement.

H3: Children’s Perceived social support moderates the relationship between drawing self-efficacy and art engagement.

**Fig 1:**
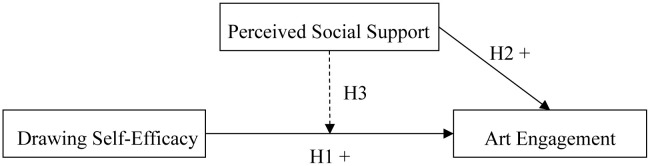
Hypothetical model is presented here for illustration. Note. Solid arrows indicate direct effects. “+” indicate positive effects. The dashed line indicates a moderation effect of Perceived Social Support on the relationship between Drawing Self-Efficacy and Art Engagement.

Fig 1 illustrates the hypothetical model.

## 3. Materials and methods

### 3.1. Research design

This study employed a quantitative, cross-sectional correlational design to examine children participating in school-based extracurricular visual arts activities. All data were collected using standardized self-report questionnaires consisting of closed-ended items. The questionnaire and methodology for this study was approved by the Human Research Ethics committee of Dhurakij Pundit University (Ethics approval number: COA006/67).

### 3.2. Description of method

The study was conducted after obtaining approval from school authorities and teachers, as well as written informed consent from both the children and their parents or legal guardians.

A pilot study was first conducted to assess the reliability and validity of the instruments. A total of 220 questionnaires were distributed, and 192 valid responses were collected. Item analysis (IA) and exploratory factor analysis (EFA) were performed. Based on the results, a refined version of the questionnaire with robust psychometric properties was finalized for the main study.

Formal data collection took place from October 15–24, 2024, during the schools’ scheduled extracurricular activity periods. Paper-based questionnaires were administered with the assistance of school teachers.

### 3.3. Sampling

The study targeted primary school children aged 10–12 years who were enrolled in school-based extracurricular visual arts activities focusing on drawing. Participation was voluntary, and written informed consent was obtained from both the children and their parents or legal guardians. A convenience sampling approach was adopted, recruiting participants from nine public primary schools in a model district in western Henan Province, China.

According to the Department of Education of Henan Province [59], approximately 9,628,800 primary school pupils were enrolled in Henan Province. The minimum required sample size was estimated using Cochran’s formula for large populations [60], with a 95% confidence level, a margin of error of 5%, and a conservative proportion estimate of 0.50. Based on these parameters, the minimum required sample size was 386 participants. To account for potential incomplete questionnaires and non-response, an over-sampling strategy was adopted.

During the formal data collection phase, 650 questionnaires were distributed, yielding 592 valid responses, corresponding to a valid response rate of 91.1%. The final sample size exceeded the minimum required threshold, ensuring adequate statistical power for subsequent analyses.

### 3.4. Research site

The study was conducted in public primary schools located in a designated after-school service model district in Henan Province, central China. Henan is situated in a region of medium economic development, where the accessibility of compulsory education is among the highest nationwide, and the allocation of educational resources is considered typical and representative [61]. Within this province, the model district serves as a pilot area for after-school services, playing a demonstrative role in the provision of extracurricular activities. Public primary schools, as the core component of China’s compulsory education system, are state-funded and government-managed. In these schools, children typically participate in at least two sessions of extracurricular visual arts activities per week, suggesting both the generalizability and representativeness of the research context.

### 3.5. Instruments

This study employs the RAISE (Arts and Humanities Engagement Scale) developed by Thapa et al. to measure art engagement [10]. The RAISE scale consists of 66 items across five dimensions: immersion, reflection, expression, acquisition, and socialization. For application to Chinese children, the scale was translated and revised, followed by expert review, rigorous item analysis, and exploratory factor analysis (EFA). As a result, 10 items were removed, and the resulting 56-item version constituted the final Art Engagement Scale employed in the current study.

The Art Engagement Scale consists of five dimensions that capture children subjective experiences and psychological involvement in school-based visual art activities. These dimensions and their corresponding structures are as follows: Acquisition: This dimension reflects the process through which children apply, develop, and refine their artistic skills. It comprises six subdimensions (Vicarious experiences, Direct encouragement, Social persuasion, Experience of mastery – Skills, Experience of mastery – Ability, Positive physiological responses). Immersion: A single-factor construct assessing the extent to which pupils feel effortlessly engaged and absorbed during art activities. Reflection: A three-factor construct measuring children cognitive and emotional reflection during participation. The subdimensions include (Reflection – Internal Life, Reflection – Internal Emotional, Reflection – External Others). Expression: A single-factor construct capturing the extent to which pupils express emotions, release feelings, and communicate ideas through art-making. Socialization: A three-factor construct assessing the social aspects of art engagement, including (Relationships, Conversation, Identity). A 5-point Likert scale was used for scoring, ranging from 1 (strongly disagree) to 5 (strongly agree), with higher scores indicating a higher subjective perception of art engagement.

This study employed the Chinese version of the Drawing Self-Efficacy Instrument (DSEI) validated by Nie and Tsai [11], based on Jaisone et al. [32]. The scale comprises 13 items across three dimensions: drawing skills (5 items; e.g., “Drawing a 2D object.”), communication expression (5 items; e.g., “Drawing to communicate ideas to others.”), and Creative expression (3 items; e.g., “Drawing something from my imagination.”). Following item analysis and exploratory factor analysis (EFA), all items were retained. A 5-point Likert scale was used, ranging from 1 (strongly disagree) to 5 (strongly agree), with higher scores indicating stronger drawing self-efficacy.

This study employs the Multidimensional Scale of Perceived Social Support (MSPSS), originally developed by Zimet et al. and later translated and revised by Yang and Han [62, 63], The scale consists of 12 items across three dimensions. After item analysis and exploratory factor analysis (EFA), two items were removed, resulting in a 10-item revised version. The final scale includes: Teacher support (4 items; e.g., “I can share my joy and sorrow with my teacher.”), Parental support (3 items; e.g., “My family always tries their best to help me.”), and Peer support (3 items; e.g., “My classmates always try their best to help me.”). A 5-point Likert scale was used, ranging from 1 (strongly disagree) to 5 (strongly agree), with higher scores indicating greater perceived social support.

### 3.6. Research procedure

Prior to data collection, researchers contacted school principals to explain the study purpose and instruments and obtained permission to conduct the study. Consent was obtained from visual arts teachers, who assisted in distributing informed consent forms to the children. Children were instructed to review the consent documents with their parents or legal guardians to decide on participation. After receiving signed consent forms from both the children and their parents or guardians, the research team verified the number of returned forms. All returned forms indicated agreement to participate. Before administering the questionnaire, researchers provided a verbal explanation of the study to the children and obtained their oral assent to ensure voluntary participation.

Researchers were present throughout the data collection process to ensure standardized administration, provide clarifications, and minimize potential misunderstandings, thereby enhancing data quality. All data were anonymized to protect participants’ confidentiality. Completed questionnaires were entered into an Excel sheet and then transferred to IBM SPSS Statistics version 22.0 for statistical analysis [64].

### 3.7. Validity and reliability

Table 1 shows the internal consistency of the study instruments. Cronbach’s α values for the overall scales ranged from.830 to.955, and for individual dimensions from.678 to.931, indicating that all measures demonstrated acceptable to excellent reliability in this sample of Chinese children [65].

**Table 1:** Reliability of the study instruments.

Construct	Dimension	Items	Cronbach’s α
Art Engagement	Overall scale	56	.955
(AE)	Immersion	3	.719
	Reflection	12	.836
	Expression	4	.836
	Acquisition	24	.931
	Socialization	13	.898
Drawing Self-Efficacy	Overall scale	13	.881
(DSE)	Drawing skills	5	.824
	Communication expression	5	.842
	Creative expression	3	.727
Perceived Social Support	Overall scale	10	.830
(PSS)	Teacher Support	4	.806
	Parental Support	3	.721
	Peer Support	3	.678

Note. *N* = 592.

To assess discriminant validity, this study adopted the criterion proposed by Fornell and Larcker [66], which compares the square root of the Average Variance Extracted (AVE) for each construct with its correlations with other constructs. Discriminant validity is considered acceptable when the square root of a construct’s AVE exceeds its correlations with all other constructs, indicating that the construct shares more variance with its own indicators than with others. In this study, all constructs met this criterion, demonstrating that the model possesses satisfactory discriminant validity. The detailed results are presented in Table 2.

**Table 2:** Results of discriminant validity analysis.

Variable	1	2	3	4	5	6	7	8	9	10	11	12	13	14	15	16	17	18	19	20
1.Tea	**.716**																			
2.Par	.392***	**.698**																		
3.Pee	.482***	.441***	**.646**																	
4.Imm	.391***	.283***	.351**	**.684**																
5.RIL	.122**	.106*	.142**	.180***	**.797**															
6.RIE	.271***	.227***	.289***	.371***	.437***	**.637**														
7.REO	.327***	.168***	.285***	.343***	.312***	.420***	**.662**													
8.EXP	.320***	.337***	.344***	.502***	.146***	.330***	.233***	**.752**												
9.AEA	.467***	.348***	.446***	.522***	.251***	.382***	.428***	.498***	**.711**											
10.AES	.280***	.310***	.297***	.455***	.169***	.325***	.267***	.495***	.545***	**.724**										
11.AVE	.364***	.247***	.337***	.413***	.261***	.364***	.490***	.420***	.593***	.486***	**.685**									
12.ADE	.357***	.251***	.326***	.496***	.230***	.328***	.310***	.458***	.524***	.582***	.499***	**.755**								
13.ASP	.436***	.368***	.428***	.444***	.201***	.342***	.373***	.483***	.572***	.408***	.489***	.522***	**.693**							
14.APPR	.354***	.266***	.354***	.442***	.053	.257***	.229***	.471***	.532***	.410***	.414***	.433***	.558***	**.822**						
15.SR	.397***	.248***	.388***	.411***	.189***	.351***	.323***	.353***	.490***	.384***	.469***	.470***	.514***	.456**	**.750**					
16.SC	.414***	.266***	.411***	.411***	.188***	.340***	.311***	.396***	.528***	.421***	.491***	.503***	.511***	.500***	.634***	**.750**				
17.SI	.366***	.305***	.348***	.443***	.273***	.414***	.387***	.499***	.557***	.500***	.495***	.495***	.555***	.513***	.524***	.551***	**.688**			
18.Ds	.165***	.188***	.185***	.300***	.136***	.168***	.253***	.250***	.360***	.315***	.313***	.371***	.304***	.264***	.297***	.308***	.312***	**.700**		
19.Com	.279***	.203***	.248***	.371***	.230***	.266***	.345***	.369***	.442***	.369***	.477***	.449***	.470***	.400***	.401***	.394***	.499***	.478***	**.723**	
20.CE	.250***	.125**	.259***	.382***	.172***	.207***	.286***	.351***	.382***	.329***	.366***	.382***	.406***	.321***	.341***	.346***	.431***	.399***	.627***	**.691**

Note. *N* = 592, Tea = Teacher support; Par = Parental support; Pee = Peer support; Imm = Immersion; RIL = Reflection-Internal Life; RIE = Reflection-Internal Emotional; REO = Reflection-External Others; Exp = Expression; AEA = Acquisition-Experience of mastery-Ability; AES = Acquisition-Experience of mastery-Skills; AVE = Acquisition -Vicarious experiences; ADE = Acquisition-Direct encouragement; ASP = Acquisition-Social persuasion; APPR = Acquisition-Positive physiological responses; SR = Socialization- Relationships; SC = Socialization-Conversation; SI = Socialization-Identity; DS = Drawing skill; Com = Conveying and communicating; CE = Creative expression. The bold values are the square root of the AVE. *p < .05, **p < .01, ***p < .001

### 3.8. Method of data analysis

Data were analyzed in IBM SPSS Statistics 22.0 [64]. Analyses comprised descriptive statistics, Pearson correlations, and hierarchical regression, with significance defined as *p* < .05 [65].

To test the hypothesized moderation model, hierarchical regression analysis was employed, with art engagement as the dependent variable, drawing self-efficacy as the independent variable, and perceived social support as the moderator. All continuous predictor variables were standardized prior to analysis to reduce potential multicollinearity [67]. In Step 1, drawing self-efficacy was entered into the regression model. In Step 2, perceived social support was added to examine its main effect. In Step 3, the interaction term between drawing self-efficacy and perceived social support was entered to test the moderation effect. Changes in explained variance (ΔR²) were examined across steps to assess the incremental contribution of each block.

Prior to regression analyses, the assumptions of hierarchical regression, including linearity, normality of residuals, homoscedasticity, and absence of multicollinearity, were examined and were adequately met. Variance inflation factor (VIF) values were all below 10, indicating no severe multicollinearity [65].

The data had a nested structure, with students clustered within schools. However, the primary interest of this study was in individual-level associations among drawing self-efficacy, perceived social support, and art engagement, and the number of schools was insufficient to support reliable multilevel modeling. Therefore, hierarchical regression analyses were conducted at the individual level.

## 4. Results

### 4.1. Demographic characteristics

The final sample comprised 592 children, including 127 boys (21.5%) and 465 girls (78.5%), which is consistent with prior evidence of gender differences in participation in extracurricular visual arts activities [39]. The age distribution indicated that most participants were 10 years old (*N* = 235, 39.7%) or 11 years old (*N* = 256, 43.2%), while participants aged 12 accounted for 17.1% of the sample. Age information was used only for descriptive reporting and excluded from the shared dataset to ensure participant confidentiality. The characteristics of the participants are presented in Table 3.

**Table 3:** Demographic characteristics of the participants.

Characteristic	Subcategory	Frequency (%)
Gender	Boys	127 (21.5)
	Girls	465 (78.5)
Age	10	235 (39.7)
	11	256 (43.2)
	12	101 (17.1)

Note. *N* = 592.

### 4.2. Descriptive statistics

Descriptive statistics for the main study variables are presented in Table 4. Drawing Self-Efficacy had a mean of 3.662 (*SD* = 0.771), Perceived Social Support had a mean of 3.969 (*SD* = 0.638), and Art Engagement had a mean of 3.727 (*SD* = 0.578). The standard deviation values ranged from 0.578 to 0.771, indicating that scores were moderately concentrated around their respective means.

**Table 4:** Descriptive statistics for main study variables.

Variable	DSE	PSP	AE
*M*	3.662	3.969	3.727
*SD*	0.771	0.638	0.578

Note. *N* = 592, *M* = Mean; *SD* = Standard Deviation; DSE = Drawing Self-Efficacy; PSP = Perceived Social Support; Art Engagement = AE.

### 4.3. Hierarchical regression analyses

To minimize the potential for multicollinearity, all constructs of the proposed research model were standardized following the recommendations of Aiken and West [67]. A hierarchical regression analysis was employed to investigate the predictor and moderator variables, specifically to assess whether social support moderates the relationship between drawing self-efficacy and art engagement. In the first step, drawing self-efficacy was entered as the predictor of art engagement. In the second step, social support was added to the model together with drawing self-efficacy to examine their main effects on art engagement. Finally, the interaction term between drawing self-efficacy and social support was included to test the moderating effect of social support on the relationship between drawing self-efficacy and art engagement.

As shown in Model 1 of Table 5, drawing self-efficacy had a significant and positive impact on art engagement (β = .612, *p* < .001), explaining 37.4% of the variance (R²). These results indicate that drawing self-efficacy can significantly and positively predict pupils’ art engagement, accounting for 37.3% of the variance. Therefore, Hypothesis H1 of this study is supported.

**Table 5:** Hierarchical regression analysis predicting art engagement.

Variable	Model 1	Model 2	Model 3	VIF
**Main effects**				
DSE	.612^***^	.464^***^	.457^***^	1.140
**Moderator**				
PSP		.447^***^	.438^***^	1.148
**Interactions**				
DSE * PSP			−.056^*^	1.057
F	353.005^***^	363.305^***^	244.749^***^	
R^2^	37.4%	55.2%	55.5%	
ΔR^2^	37.4%	7.8%	0.3%	
AdjR^2^	37.3%	55.1%	55.3%	

Note. *N* = 592, DSE = Drawing Self-Efficacy; PSP = Perceived Social Support; VIF = Variance Inflation Factor; F = F statistic; R² = Coefficient of determination; ΔR^2^ = Change in R-squared from the previous step; AdjR² = Adjusted coefficient of determination. *p < .05; ***p < .001.

As shown in Model 2 of Table 5, Perceived social support has a significant and positive impact on art engagement (β = .447, *p* < .001), explaining 55.2% of the variance (R²). These results indicate that Perceived social support can significantly and positively predict pupils’ art engagement, accounting for 55.1% of the variance. Therefore, Hypothesis H2 of this study is supported.

As shown in Model 3 of Table 5, perceived social support exerted a significant negative moderating effect on the relationship between drawing self-efficacy and art engagement (β = –.056, *p* < .05). This finding suggests that higher levels of perceived social support weaken the positive relationship between drawing self-efficacy and art engagement. Model 3 explained 55.5% of the variance in art engagement (R² = 55.5%, AdjR² = 55.3%), and the inclusion of the interaction term accounted for an additional 0.3% of the variance (ΔR² = 0.3%, *p* < .05; see Table 5), indicating a small but meaningful moderation effect. Therefore, Hypothesis H3 of this study is supported. Additionally, with VIF < 10, there is no severe collinearity problem.

**Fig 2:**
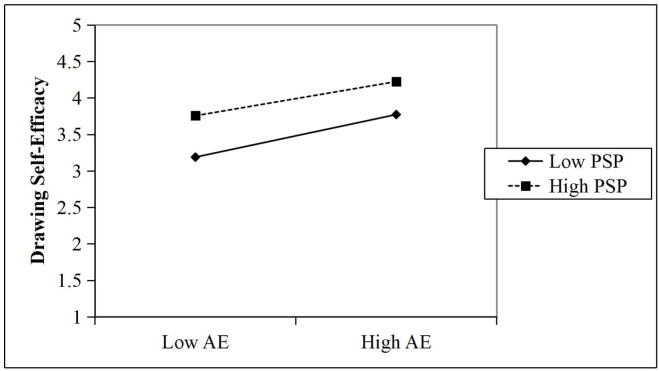
Moderating effect diagram. Note. PSP = Perceived Social Support; AE = Art Engagement. “High” and “Low” levels of PSP and AE represent values one standard deviation above and below the mean, respectively.

Fig 2 shows the graphical representation of the moderating effect of perceived social support, which weakens the positive relationship between drawing self-efficacy and art engagement. For illustration purposes, simple slopes were plotted at one standard deviation above and below the mean of perceived social support, following the procedure proposed by Aiken and West [67]. All variables were mean-centered prior to analysis.

## 5. Discussion

### 5.1. The relationship between drawing self-efficacy and art engagement

This study found that drawing self-efficacy has a significant positive impact on art engagement, indicating that children’s perception of their own drawing ability and their active participation in artistic activities play a crucial role in shaping their artistic experiences. Drawing self-efficacy reflects an individual’s confidence in, and recognition of, their artistic abilities, which in turn influences their cognitive, emotional, and behavioral investment in drawing activities. These findings are consistent with the qualitative research of Hickman and Lord and Moorefield Lang, which demonstrated a close relationship between self-efficacy and children’s art engagement [16,19], and further empirically verify that children with higher self-efficacy exhibit more positive engagement in artistic practice [8]. In addition, these results align with the core concepts of Cognitive Evaluation Theory [55]. When autonomy and competence are satisfied in the context of drawing, children are more likely to participate in artistic activities based on intrinsic interest, thereby enhancing their art engagement. This highlights the value of cultivating drawing self-efficacy as a key skill, suggesting that promoting positive self-efficacy is essential for improving the quality of children’s engagement in drawing activities.

From a curriculum design perspective, this finding offers practical guidance. Teachers should pay attention to children’s performance in three areas: drawing skills, communication expression, and creative expression [11,20]. They should provide feedback that is specific and constructive, and design tasks that match children’s current abilities while also incorporating reasonable challenges [9,51]. Such experiences allow children to experience success more frequently, gradually building their drawing self-efficacy, which in turn helps sustain long-term art engagement.

### 5.2. The relationship between perceived social support and art engagement

This study found that perceived social support has a significant positive impact on art engagement. Children who receive greater support from parents, teachers, and peers in drawing activities are more likely to develop artistic skills, express emotions, reflect on life experiences, focus on self-expression, and engage in positive social interactions [35]. These findings are consistent with Cameron et al., who reported that parental and teacher support for children’s drawing significantly influences their artistic development and self-expression [43]. Moreover, this study suggests that social support not only facilitates skill development but also enhances children’s emotional experiences and social interactions in artistic practice, thereby expanding the dimensions of art engagement. Additionally, the results align with Çetin’s findings, which indicate that increased peer interaction in artistic activities promotes artistic communication and allows children to experience the intrinsic value of artistic practice more deeply [6].

These findings can be explained by Cognitive Evaluation Theory. When children feel supported by teachers, parents, or peers in drawing activities, these external factors satisfy basic psychological needs such as autonomy, competence, and relatedness, enhancing intrinsic motivation and further increasing art engagement [55]. Specifically, positive feedback from teachers and encouragement from parents strengthen children’s sense of competence and autonomy, while peer support reinforces motivation through social connection and cooperation [68, 44]. Overall, the results emphasize the pivotal role of social support as an external factor in children’s artistic behavior and emotional-cognitive development [8,26]. Providing positive social support in school and family environments can enhance emotional engagement and social interaction in drawing activities, thereby improving the overall art participation experience.

From an educational policy perspective, this finding aligns with the “Double Reduction” policy [69], which has created more time and space for after-school services [57,70], and highlights the importance of art education in society, increasing social support for children’s participation in drawing activities.

From a curriculum perspective, schools and teachers should provide a safe and open environment for artistic practice [71, 72], enabling children to explore freely, take risks, and express themselves. Art activities should emphasize their social nature. Group drawing, peer sharing, and joint exhibitions strengthen peer connections [70,73], fostering belonging and cooperation. Together, these factors enhance intrinsic motivation and deepen children’s engagement in art.

### 5.3. The negative moderating effect of perceived social support

This study found that perceived social support negatively moderates the relationship between drawing self-efficacy and art engagement. Specifically, higher levels of perceived social support reduce the positive impact of drawing self-efficacy on art engagement, suggesting that excessive support may weaken its benefits. Henderlong and Lepper reported that excessive external rewards or social support can undermine children’s intrinsic motivation [74],thereby reducing autonomy. Similarly, Hiçyılmaz noted that continuous praise may increase external motivation but weaken interest in the activity itself [26]. According to Cognitive Evaluation Theory, excessive external support may lead children to attribute their engagement to external rewards or social recognition rather than internal interest and enjoyment, which can diminish the influence of drawing self-efficacy. In such contexts, children may focus more on social feedback and less on improving their drawing skills, thereby reducing art engagement.

In China, this phenomenon may be particularly pronounced due to limited school art resources and insufficient teacher expertise [69,73,75], coupled with misalignment between parental and teacher support goals and children’s needs [8]. Excessive encouragement or intrusive guidance can constrain autonomy and foster dependence on external recognition, further weakening art engagement [46,76].

Beyond Cognitive Evaluation Theory, this moderating effect may reflect a motivational substitution mechanism [77]. When pupils perceive high social support, extrinsic factors may become the primary driver of engagement, reducing the relative importance of intrinsic motivators such as drawing self-efficacy. This suggests a structural shift in motivational sources, where initial intrinsic motivation is gradually replaced by extrinsic reinforcement from parents, teachers, or peers.

This phenomenon underscores the inherently social nature of drawing activities [7, 8]. Perceived social support enable pupils to fulfill their relational needs, which enhances their overall participation motivation and consequently promotes deeper engagement in artistic pursuits. However, under such conditions, the marginal effect of drawing self-efficacy, which is rooted in autonomy and competence beliefs, may be significantly weakened.

These findings highlight the complexity of motivational dynamics in school-based drawing activities. Effective engagement depends on both children’s self-directed beliefs in their abilities and sustained, appropriately calibrated social support. Different psychological needs contribute through distinct mechanisms, providing a theoretical foundation for future studies on the interplay of intrinsic and extrinsic motivation.

Cultural context may also influence this effect. In educational systems that emphasize parental and teacher authority, as in many East Asian contexts, excessive support is more likely perceived as controlling, undermining autonomy [46,72]. Conversely, in systems emphasizing learner independence, the same support may not produce as strong a negative effect [58]. Comparative research could clarify these cultural contingencies.

At a practical level, enhancing teacher training may mitigate the risks of excessive support. Teachers who can differentiate between constructive encouragement and controlling intervention can provide feedback that strengthens children’s competence without reducing autonomy [71,73]. Such professional development ensures that social support facilitates, rather than constrains, engagement in drawing activities.

## 6. Conclusion

This study examined the relationship between drawing self-efficacy and art engagement, with particular attention to the moderating role of perceived social support. The findings revealed a significant positive association between drawing self-efficacy and art engagement, indicating that children’s confidence in their drawing ability serves as a key internal driver of their participation in drawing activities. Notably, perceived social support was found to exert a negative moderating effect on this relationship, such that high levels of perceived social support weakened the positive influence of drawing self-efficacy on art engagement.

This counterintuitive pattern may be explained by a process of motivational redistribution. When social support is excessive, children may rely more heavily on external validation, diminishing the motivational impact of their own drawing self-efficacy. Rather than strengthening intrinsic motivation, overly intensive support can foster externally regulated forms of engagement. These findings challenge the common assumption that social support is always beneficial and highlight the importance of considering its quality and intensity. They contribute to a more nuanced understanding of art engagement by demonstrating how individual motivational beliefs and social contextual factors interact in shaping children’s participation in drawing activities.

The results also have important implications for the Chinese educational context, where school-based drawing programs often face limited resources, facilities, and professional development opportunities. In such settings, well-intentioned support may unintentionally become over-guidance, potentially undermining pupils’ autonomy. To promote sustained engagement, educators and parents should prioritize autonomy-supportive practices, including encouraging exploration, offering meaningful choices, and affirming children’s independent decisions and creative processes. At the institutional level, greater investment in professional development for drawing educators can help create learning environments that support student-centered artistic experiences and foster long-term engagement.

## 7. Limitations and recommendations for future research

While this study provides valuable insights into the relationship between drawing self-efficacy, perceived social support, and art engagement, several limitations should be noted. First, the exclusive reliance on children’s self-reports may introduce biases related to social desirability or subjective perception. Second, the sample was restricted to primary school children within Henan Province’s after-school service demonstration zones, limiting the ecological validity and generalizability of the findings. Third, the study did not examine in depth the direct effect of perceived social support on art engagement or the specific conditions under which its negative moderating effect occurs. Finally, motivation-related dynamics, including attentional lapses or emotional variability, were not accounted for, although these factors may significantly influence engagement and self-efficacy, as even initially motivated pupils can experience fluctuations due to situational or emotional factors.

To address these limitations, future research should consider expanding to larger and more diverse samples across different cultural and educational contexts to enhance generalizability. Incorporating multi-informant perspectives, such as teachers or parents, and combining observational or experimental measures with self-reports can reduce bias and provide a more comprehensive assessment of children’s engagement. Further studies could also explore additional moderating or mediating variables, including teacher training quality, access to drawing resources, or school policy support, to deepen understanding of the mechanisms underlying art engagement. Moreover, examining motivation-related dynamics, such as fluctuations in interest, attention, and emotional states, would offer more nuanced insights into how children sustain engagement in school-based drawing activities over time.

## Supporting information

S1 Appendix Study questionnaire. This file contains the full questionnaire used in the study, including validated measures of perceived social support, drawing self-efficacy, and art engagement. https://figshare.com/articles/dataset/___/29380391?file=55918775.
(DOCX)

S2 Appendix Dataset. This file includes the cross-sectional survey data collected from 592 pupils (aged 10–12) in Henan Province, China. All participants were engaged in school-based extracurricular visual arts activities. https://figshare.com/articles/dataset/___/29380391?file=55918742.
(CSV)
