# Supplementary material for: Too much of a good thing? The moderating role of children’s perceived social support in drawing activities
Source: PLoS One. 2026 Feb 17;21(2):e0330470. doi: 10.1371/journal.pone.0330470 (PMC12912555; doi:10.1371/journal.pone.0330470)
Supplement: S1 Appendix — This file contains the full questionnaire used in the study, including validated measures of perceived social support, drawing self-efficacy, and art engagement. https://figshare.com/articles/dataset/___/29380391?file=55918775. (DOCX) [file pone.0330470.s001.docx]

**S1 Appendix_Questionnaire**

**Part 1:** P**erceived Social Support Scale**

| Reflecting on your participation in school-based extracurricular visual arts activities (art club classes), please indicate to what extent the following statements align with your actual experience. | Strongly Disagree | Disagree | Neutral | Agree | Strongly Agree |
| --- | --- | --- | --- | --- | --- |
| Tea-1. When I need help, my teacher will be by my side. |  |  |  |  |  |
| Tea-2. I can share my joy and sorrow with my teacher. |  |  |  |  |  |
| Par-3. My family always tries their best to help me. |  |  |  |  |  |
| Par-4. My family provides me with the emotional help and support I need. |  |  |  |  |  |
| Tea-5. When I have difficulties, my teacher can comfort me. |  |  |  |  |  |
| Pee-6. My classmates always try their best to help me. |  |  |  |  |  |
| Par-8. I can talk to my family about the problems I encounter. |  |  |  |  |  |
| Pee-9. I have classmates with whom I can share my joys and sorrows. |  |  |  |  |  |
| Tea-10. During activities, my teacher cares about my feelings. |  |  |  |  |  |
| Pee-12. I can confide in my classmates about the problems I encounter. |  |  |  |  |  |

*Note:* Tea=Teacher support; Par=Parental support; Pee=Peer support

**Part 2: Arts Engagement Scale**

| Thinking about when you engage in school-based extracurricular visual art activities (art club classes), please indicate the extent to which you agree with the following statements - that is, the average level of participation in the activities. | | Strongly Disagree | | Disagree | | Neutral | | Agree | | Strongly Agree | |
| --- | --- | --- | --- | --- | --- | --- | --- | --- | --- | --- | --- |
| **Immersion** | | | | | | | | | | |  |
| ***When I engage in extracurricular visual arts activities...*** | | | | | | | | | | |  |
| Imm-4.During the activity, I can easily focus all my attention. |  | |  | |  | |  | |  | |  |
| Imm-5.Once the activity starts, I can easily engage throughout. |  | |  | |  | |  | |  | |  |
| Imm-6. I tend to set aside life's problems during the activity. |  | |  | |  | |  | |  | |  |
| **Reflection** | | | | | | | | | | |  |
| ***When I engage in extracurricular visual arts activities...*** | | | | | | | | | | |  |
| RIL-7. I ask myself some life questions. |  | |  | |  | |  | |  | |  |
| RIL-8. I ponder the meaning of life. |  | |  | |  | |  | |  | |  |
| RIL-9. I reflect on my own life. |  | |  | |  | |  | |  | |  |
| RIE-12. I savor past experiences. |  | |  | |  | |  | |  | |  |
| RIE-13. I take time to relish past joyful feelings. |  | |  | |  | |  | |  | |  |
| RIE-14. I cherish the various experiences in my life. |  | |  | |  | |  | |  | |  |
| RIE-15. I revisit past experiences. |  | |  | |  | |  | |  | |  |
| REO-17. I try to understand things through others' perspectives. |  | |  | |  | |  | |  | |  |
| REO-18. I attempt to adopt other people's viewpoints. |  | |  | |  | |  | |  | |  |
| REO-19. I ponder how others think. |  | |  | |  | |  | |  | |  |
| REO-20. I strive to comprehend others' positions. |  | |  | |  | |  | |  | |  |
| REO-21. I make an effort to see the world through others' eyes. |  | |  | |  | |  | |  | |  |
| **Expression** | | | | | | | | | | |  |
| ***When I engage in extracurricular visual arts activities...*** | | | | | | | | | | |  |
| Exp-22. I can express my pursuits firmly without being influenced by others. |  | |  | |  | |  | |  | |  |
| Exp-23. I feel like I am being true to myself. |  | |  | |  | |  | |  | |  |
| Exp-24. I find it easy to show my true self. |  | |  | |  | |  | |  | |  |
| Exp-25. I can freely express my thoughts and emotions. |  | |  | |  | |  | |  | |  |
| **Acquisition** | | | | | | | | | | |  |
| ***When I engage in extracurricular visual arts activities...*** | | | | | | | | | | |  |
| AEA-27. I develop new skills. |  | |  | |  | |  | |  | |  |
| AEA-28. I learn new abilities. |  | |  | |  | |  | |  | |  |
| AEA-29. I establish new competencies and capabilities. |  | |  | |  | |  | |  | |  |
| AEA-30. I gain experiences to learn new skills. |  | |  | |  | |  | |  | |  |
| AEA-35. I observe successful people and learn how to act correctly. |  | |  | |  | |  | |  | |  |
| AEA-36. By learning from others' failures, I gain confidence when facing similar issues. |  | |  | |  | |  | |  | |  |
| AES-31. I feel competent. |  | |  | |  | |  | |  | |  |
| AES-32. Generally, I feel I'm doing well. |  | |  | |  | |  | |  | |  |
| AES-33. I feel a sense of accomplishment. |  | |  | |  | |  | |  | |  |
| AES-34. I feel successful. |  | |  | |  | |  | |  | |  |
| AVE-37. Observing others motivates me to do better. |  | |  | |  | |  | |  | |  |
| AVE-38. I can imagine my behavior being similar to those around me. |  | |  | |  | |  | |  | |  |
| AVE-39. Through observing others, I discover new ways of handling things. |  | |  | |  | |  | |  | |  |
| ADE-40. Others praise my skills. |  | |  | |  | |  | |  | |  |
| ADE-41. Others say I am good at this. |  | |  | |  | |  | |  | |  |
| ADE-42. Others say I am talented. |  | |  | |  | |  | |  | |  |
| ADE-43. I am praised for my abilities and potential. |  | |  | |  | |  | |  | |  |
| ADE-44. My abilities are recognized. |  | |  | |  | |  | |  | |  |
| ASP-46. Others' opinions help me improve. |  | |  | |  | |  | |  | |  |
| ASP-48. I always receive timely assistance. |  | |  | |  | |  | |  | |  |
| ASP-49. I have found my supporters. |  | |  | |  | |  | |  | |  |
| APPR-50. I feel relaxed and calm. |  | |  | |  | |  | |  | |  |
| APPR-51. I feel free. |  | |  | |  | |  | |  | |  |
| APPR-52. I feel at ease. |  | |  | |  | |  | |  | |  |
| **Socialization** | | | | | | | | | | |  |
| ***Engaging in extracurricular visual arts activities allows me to...*** | | | | | | | | | | |  |
| SR-53. Strengthening interpersonal relationships. |  | |  | |  | |  | |  | |  |
| SR-54. Building deep relationships with others. |  | |  | |  | |  | |  | |  |
| SR-55. Maintaining close relationships with others. |  | |  | |  | |  | |  | |  |
| SR-56. Establishing warm and trusting relationships with others. |  | |  | |  | |  | |  | |  |
| SR-57. Feeling closer to people in my life. |  | |  | |  | |  | |  | |  |
| SC-58. Engaging in more meaningful conversations. |  | |  | |  | |  | |  | |  |
| SC-59. Discussing more interesting topics with others. |  | |  | |  | |  | |  | |  |
| SC-60. Engaging in deeper conversations with others. |  | |  | |  | |  | |  | |  |
| SI-62. Beginning to understand what kind of person I am. |  | |  | |  | |  | |  | |  |
| SI-63. Exploring new roles and interests that are important to me. |  | |  | |  | |  | |  | |  |
| SI-64. Better understanding who I am. |  | |  | |  | |  | |  | |  |
| SI-65. Seeing myself more accurately. |  | |  | |  | |  | |  | |  |
| SI-66. Being satisfied with my identity and roles. |  | |  | |  | |  | |  | |  |

*Note:* Imm=Immersion; RIL=Reflection-*Internal Life*; RIE=Reflection-*Internal Emotional*; REO=Reflection-*External Others*; Exp=Expression; AEA=Acquisition-*Experience of mastery-Ability*;

AES=Acquisition-*Experience of mastery-Skills*; AVE=Acquisition -*Vicarious experiences*; ADE=Acquisition-*Direct encouragement*; ASP=Acquisition-*Social persuasion*; APPR=Acquisition-*Positive physiological responses*; SR=Socialization- *Relationships*; SC=Socialization-*Conversation*; SI=Socialization-*Identity*;

**Part 3: Drawing Self-Efficacy Scale**

| Please evaluate your drawing ability based on the following statements. | **1** | **2** | **3** | **4** | **5** |
| --- | --- | --- | --- | --- | --- |
| DS-1. Drawing a 2D object |  |  |  |  |  |
| DS-2. Drawing a 3D object |  |  |  |  |  |
| DS-3. Drawing a product |  |  |  |  |  |
| DS-4. Drawing a vehicle |  |  |  |  |  |
| DS-5. Drawing a building |  |  |  |  |  |
| Com-6. Drawing when I am under pressure to come up with an idea |  |  |  |  |  |
| Com-7. Drawing to explain or teach a concept to others |  |  |  |  |  |
| Com-8. Drawing to communicate ideas to others |  |  |  |  |  |
| Com-9. Drawing to generate creative ideas for a project |  |  |  |  |  |
| Com-10.Drawing to think through a problem |  |  |  |  |  |
| CE-11.Drawing something from my imagination |  |  |  |  |  |
| CE-12. Drawing to express myself |  |  |  |  |  |
| CE-13. Drawing a person |  |  |  |  |  |

*Note:* DS=Drawing skill; Com=Conveying and communicating; CE=Creative expression
